# Practical wisdom in medicine: defending a multidimensional, integrated view of an indispensable virtue

**DOI:** 10.1007/s11017-026-09760-7

**Published:** 2026-05-23

**Authors:** Lauris C. Kaldjian, Kristján Kristjánsson, Shane McLoughlin

**Affiliations:** 1 Jubilee Centre for Character and Virtues, University of Birmingham, Birmingham, UK; 2 Program in Bioethics and Humanities, Carver College of Medicine, University of Iowa, 1-106 MEB, 500 Newton Road, 52242 Iowa City, USA

**Keywords:** Practical wisdom (phronesis), Aristotelian standard model, Phronesis eliminativism, Virtue ethics, Medicine, Network analysis

## Abstract

By defending practical wisdom (*phronesis*) as a multidimensional virtue that complements the moral virtues, we offer a critique of “practical wisdom eliminativism”, with special attention to the context of medicine, arguing that the core dimensions of practical wisdom enjoy broad consensus. As the meta-virtue that demonstrates excellence in ethical decision-making, practical wisdom recognizes and employs the best means to achieve good and worthwhile ends by integrating goals, perception of context, moral virtues, deliberation, reason-guided emotion, and motivation. In medicine, practical wisdom encompasses patient-centered deliberation directed toward ends of health and flourishing that promote the patient’s good. To counter the notion that practical wisdom is a redundant concept (the eliminativist view), we provide philosophical arguments, evidence from medical practitioners, and psychometric data from a detailed empirical study of US and UK adults. Practical wisdom has survived for more than 2300 years as a unified and unifying intellectual meta-virtue that guides the moral virtues. We believe the reasons we put forward explain why *phronesis* should be expected to endure as a meaningful, multidimensional concept reflecting the nature of moral deliberation in response to practical challenges in life and medicine.

## Introduction

The simplest definition of practical wisdom (*phronesis*) is excellence in ethical decision-making [1]. As an intellectual meta-virtue with a unique task, it is typically considered to have a more specific “hands-on” role than general wisdom [2]. Practical wisdom arguably develops later than the moral virtues (such as honesty and loyalty), but it cannot be expressed without them. More specifically, once developed (presumably during adolescence and after), it attaches itself to the moral virtues and helps us adjudicate how best to exhibit those moral virtues in the rough and tumble of personal or professional life, especially when different moral virtues are relevant to the same situation but seem to come into conflict with one another. For example, should a physician immediately ‘whistle-blow’ on a close colleague whom she suspects of showing up intoxicated at work, or should the first response be to have a private ‘personal touch’ conversation with the colleague and try to make him mend his ways?

*Phronesis* has been undergoing a significant academic revival of late. What we could call the new ‘*phronesis* bandwagon’ was originally driven by philosophers, but this interest has also percolated to psychologists, educationists, and professional ethicists. For present purposes, the most relevant development is the proliferation of studies of *phronesis* within medical practice [3–5], a development which aligns with a growing disillusionment toward deontologically grounded formal codes of ethics, as well as a surge of interest in non-codifiable virtue ethical approaches, with a focus on the objective flourishing (*eudaimonia*) of medical practitioners rather than merely their subjective wellness [6]. Despite all this growing interest, prior to the work of the Jubilee Centre at the University of Birmingham, there existed no psychologically credible operationalization of *phronesis* or an instrument to measure it [7, 8], as distinct from various existing instruments to measure wisdom on a more general psychological understanding.

To fulfill its role adequately, practical wisdom must arguably perform multiple functions simultaneously or in rapid succession [1]. The first putative function is *moral perception*: one must notice that a moral issue has been elicited and grasp, at least broadly, which moral virtues are at stake. The second function is *emotional regulation*: one must infuse one’s emotions with adequate reason so that they do not run amok and lead one astray. The third function is *moral identity*: one must possess some general blueprint of who one wants to be (as individual moral agents and as professionals) and of what one considers a good life for oneself and others. The fourth and final function is *moral adjudication*: one must be able—through the use of reason and deliberation—to synthesize and integrate the demands of the various moral virtues and to reconcile them in cases of conflict.

Unlike the moral virtues, which are typically considered to have a golden-mean architectonic between extremes of excess and deficiency, practical wisdom as an intellectual virtue only has a deficiency form. It does not make sense to say that someone has too much wisdom. That said, practical wisdom is a scalar concept—one can possess more or less of it on a sliding scale—and it is a threshold concept in the sense that someone could be considered to have sufficient practical wisdom to count as wise, although he or she does not possess perfect wisdom.

The contours of *phronesis* stated above represent what philosopher Christian Miller calls the “Standard [Aristotelian] Approach” to practical wisdom or simply “the standard model” [9]. Despite various departures from this approach by various theorists, the contemporary discourse on *phronesis* cannot escape its moorings in Aristotle’s moral philosophy; and most prominent models of late—even those designed by theorists who avoid any deference to Aristotle—bear some similarity to the four-function standard model summarized above. For example, the model of *phronesis* in medicine by Kaldjian [3] contains five functions, three of which can easily be conceptualized as sub-functions of the moral identity/blueprint function; and while Kaldjian does not include emotion regulation as a function of *phronesis*, he adds it as one of its necessary associate virtues. Non-standard models do exist, such as the so-called Aretai monistic model, which sees the moral virtues, so to speak, as outgrowths of *phronesis* rather than vice versa [10], or a MacIntyrean model that replaces flourishing as a grounding concept with decision-making skills developed within discrete social “practices” [11]. Nevertheless, even those models are heavily influenced by Aristotle’s way of thinking about the intellectual virtue in question and how it needs to perform and coordinate multiple interrelated functions.

But what if this whole way of thinking about excellence in ethical decision-making is misguided, chimerical even, and that there is no usefully invoked and specifiable discrete construct like *phronesis*? This is the critical question posed by Miller’s suggestion of *phronesis* eliminativism, i.e., that *phronesis* is a theoretically redundant concept [9, 12]. To clarify, another way to understand Miller is that he is making a simple claim about the potential non-reality of *phronesis* as a single psychological trait, similar to the suggestion made by another *phronesis* eliminativist, Dan Lapsley, who cheekily remarks that *phronesis* may turn out to be non-existent in the same way as ether (in physics) is [13]. However, in that case Miller would be fighting straw men, for no advocate of the standard model has, to the best of our knowledge, posited *phronesis* as a fully unique psychological entity (i.e., a discrete trait that is functionally separate from other known traits). The idea is rather that in order to understand how excellent ethical decision-making works, it is helpful to collate a number of co-operating functions under a single conceptual umbrella and understand it theoretically as a complex intellectual virtue. That said, this theoretical move will of course not work unless the presumed virtue explains behavioral regularities and predicts relevant outcomes synergistically over and above the individual ingredients—and that can in the end only be established through empirical inquiry, as we explain in the fourth section below.

Our purpose in the present article is fourfold: We propose to defend practical wisdom as: (1) a virtue with multiple dimensions that is not reducible to those several dimensions; (2) a virtue that complements rather than replaces the moral virtues, and (3) a virtue whose core dimensions enjoy broad consensus and can be empirically verified. The fourth and most immediate aim is to take up the challenge of ‘practical wisdom eliminativism’, especially regarding the role of practical wisdom in medicine. In doing so we address and highlight evidence for consensus regarding core dimensions of practical wisdom. One of the present authors has previously [14] responded to the 2021 version of Miller’s theoretical redundancy thesis [12] and Lapsley’s complementary psychological redundancy thesis [13]. While the present article will by necessity draw on some of the same argumentative arsenal, we invoke new arguments and evidence that we hope will move the *phronesis* discourse forward—paying special attention to its implications for the practice of medicine.

In this article we will not respond to all possible challenges regarding the conceptual validity of practical wisdom. For example, we will mostly evade deep underlying questions about moral ontology: objectivist, constructivist, or nihilistic. Nevertheless, we believe it is important to offer a few clarificatory comments about the objectivity of the ends of medicine in the following section before considering the multidimensionality of practical wisdom and grounds for consensus about its core dimensions.

## Ends and means in practical wisdom in medicine

Miller offers a multi-pronged critique of the notion of practical wisdom in his 2025 paper [9]. Before delving into its most compelling challenges, it is important to address his references to the ends and means of medicine, as these comments set his new paper apart from his earlier and more general introduction of *phronesis* eliminativism [12]. In this more recent work, Miller briefly suggests that, in the context of medicine, general ends could include preventing suffering and promoting well-being, and he acknowledges that these very broad ends require further specification with respect to a particular patient’s suffering and need for information about risks associated with particular treatment options. We appreciate these general points but believe more ought to be said about the role played by ends and means in practical wisdom, both in general and in the particular context of medicine.

The *telos* inherent to the function of practical wisdom is meant to provide its ends-orientation, i.e., practical wisdom allows us to aim at *ends* worth pursuing (i.e., good ends) and see how these ends need to guide consideration of the *means* most likely to achieve those ends. The relationship between ends and means in the particular circumstances of life is therefore centrally important to the function of practical wisdom. This relationship traces back to Aristotle, who maintained that “virtue makes us aim at the right target, and practical wisdom makes us use the right means” [15].

Speaking generally, the ultimate end toward which practical wisdom aims is human flourishing. While visions of flourishing are contested in morally pluralistic societies [16], there are more proximate (and less contested) ends in our lives toward which practical wisdom also allows us to aim. In medicine, the more proximate end is health [17], and even more proximate than health are goals of care, such as curing, living longer, improving function, or enhancing comfort [18]. Even though particular concepts of health may be contested [17, 19], the idea that health serves as a *telos* more proximate and domain-specific than flourishing is clear. In this way, practical wisdom in medicine illustrates how end-setting and end-specification are inherent to the deliberative function of this virtue.

**Fig. 1:**
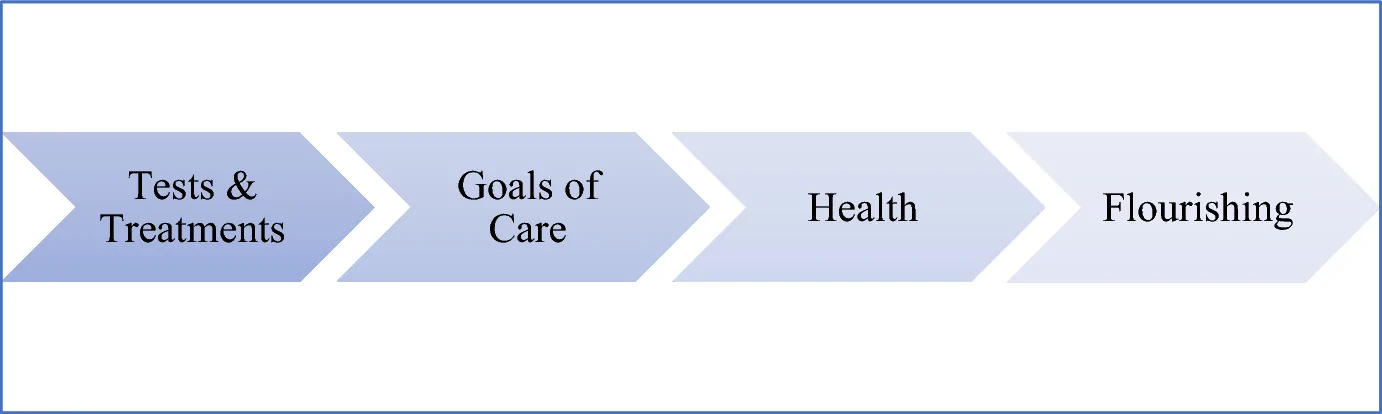
The trajectory of intentionality in healthcare

It is interesting that Miller’s discussion of end-setting for practical wisdom in medicine [9] does not refer to health as medicine’s central end (instead, as noted above, he considers general ends of preventing suffering and promoting wellbeing). This absence neglects a potent illustration (health as the end of medicine) of the teleological orientation that is a core dimension of practical wisdom, one which (at least when health is defined biostatistically) can provide an objective grounding for practical decision-making within a morally realist framework of medical ethics. Regarding end-specification in the context of medicine, we believe Miller’s concerns could be alleviated, if not resolved, by giving more attention to the teleological orientation of the practice of medicine and, thereby, to the power of medicine to illustrate this core dimension of practical wisdom. By attending to goals of care within the trajectory of intentionality in healthcare, one can see how the things done (treatments/tests) move toward proximate ends (goals of care), and then to a penultimate end (health) conducive to an ultimate end (flourishing), as shown in Fig. 1.

This observation suggests Miller’s account would benefit from more attention to teleology in the conceptualization of practical wisdom in medicine. Such reconfiguration would be particularly useful for those like Carr [20] who worry that “human flourishing” is too vague and underdetermined an ideal to serve as the unique objective end of a meta-virtue like *phronesis*.

As noted above, we recognize that normative concepts of health may vary—and not only among scholars but also in the minds of patients and healthcare professionals. For example, an empirical study of nearly 2000 adults in the United States found that health carries different meanings for different people [21]. The investigators categorized these meanings in four domains: (1) health depends on *physical* criteria (bodily and biomedical criteria); (2) health depends on *psychosocial* criteria (mental, emotional, spiritual and social, including self-esteem and self-concept); (3) health depends on the possession of *capacity* (being enabled to live an active life); and (4) health depends on taking of *control* (demonstrating the self-control needed to enact healthy behaviors). These four types of meaning map reasonably well onto two of the more prevalent theoretical accounts of health (biological concepts vs. enablement or well-being concepts): i.e., the study’s first domain (health as a matter of physical criteria) reflects the biostatistical theory of Boorse [19], whereas all four of the study’s domains pertain generally to, for instance, the holistic theory of Nordenfelt [17].

Physicians are trained to rely on the biostatistical concept of health to assess problems patients bring to clinical encounters in order to determine whether or not these problems will be treated as medical problems. This biostatistical assessment forms part of shared decision making in which a physician and patient “relate to and influence each other as they collaborate in making decisions about the patient’s health care” [22, p. 277] But it is also important, of course, to acknowledge that medical decisions need the patient-centeredness that is supported by holistic approaches that take seriously the patient’s beliefs about health and well-being. Consider a routine clinical example: a patient has high cholesterol levels and the physician recommends a statin medication to lower the cholesterol level and decrease the patient’s risk of a stroke or heart attack; if the patient experiences no loss of well-being and does not believe high cholesterol is a sufficiently serious problem, it is unlikely the patient will be persuaded to follow the physician’s recommendation. Consider also a much more dangerous and vexing clinical example: a patient has anemia that is rapidly worsening and the physician urgently recommends a blood transfusion because the severity of blood loss will soon (with a high degree of likelihood) be incompatible with life; if the patient has firm religious beliefs about flourishing that prioritize spiritual well-being above physical survival and prohibit the use of blood transfusions, it is unlikely the patient will be persuaded to follow the physician’s recommendation, even if death is the predictable and preventable outcome.

These brief examples demonstrate how the idea of the patient’s good has multiple components encompassing a medical good, the patient’s sense of their own personal good, an assessment of the human good, and a spiritual good [23]. These components reflect the bio-psycho-socio-spiritual dimensions of human beings, the ways in which different goods conduce to health and flourishing, and the potential for tensions between different goods which may require prioritization in order to be reconciled. Such considerations emphasize the benefit of hybrid or pluralist concepts of health that combine standards of objective functioning and elements of subjective well-being [24]. On such views, healthcare can be seen as a process of shared decision making that is simultaneously guided by, on the one hand, goals grounded in the givenness of human biology, with its capacities and limitations and, on the other hand, the beliefs, values, and priorities of patients as moral agents [18].

Further consideration of the practice of medicine might also help Miller better appreciate the way ends and means are interrelated and justified. Miller refers to a “justification function” of practical wisdom by which good reasons justify choices among possible ends. As an example in healthcare, he offers the end of truthfulness (“not deceiving patients”) based on respect for the dignity of persons. But his remarks do not give the reader a full enough sense of the teleologically meaningful landscape which a virtue-based (practical-wisdom-guided) account entails. Instead, Miller appears silent on the way teleology invites one to see that practical wisdom is fundamentally a matter of deliberating and acting toward the end of the specific activity in view, in this case the end of health and healing. This end then needs to be placed within a broader ethical framework that includes moral virtues and principles that carry their own moral substance, such as the virtue of honesty or the principle of truth-telling. Miller’s example of truthfulness seems to blur the difference between the purpose of a medical action (health, healing) and the obligation of a particular moral virtue (honesty). While these two features of the moral practice of medicine are related, they ought to be placed in proper alignment so that practical wisdom in medicine fixes its eyes on the *telos* of health in the process of discerning what means to that end are compatible with honesty and other moral virtues. This kind of deliberation integrates means, ends, and various moral commitments within a larger ethical framework, providing the basis for a physician’s ‘blueprint’ (moral identity). Practical wisdom allows a physician to integrate goals of care into this larger framework through the reasoning and adjudication of practical wisdom which entails moral seriousness about ends *and* means. In doing so, practical wisdom provides the ability to discern how, when, and where to express the virtue of honesty.

Generally speaking, it seems uncontroversial to suggest that Aristotelian accounts broadly agree that practical wisdom is fundamentally teleological as it concerns ends and means [25]. One can appreciate this point of consensus by the succinct statement that practical wisdom is the ability to identify the best means to good and worthwhile ends [26]. This ethically-framed, ends-and-means-integrating articulation of excellence in moral deliberation suggests there are reasonable grounds for claiming that some of the core dimensions of practical wisdom that enjoy broad consensus pertain to goal-orientation, deliberation, and ethical integration within a virtue ethics framework.

## Multidimensionality, integration, and unity in practical wisdom

Two of the most compelling challenges to the standard Aristotelian model posed by Miller’s 2025 article are the Arbitrariness Challenge (which claims that lists of the functions of *phronesis* differ in arbitrary ways, even among proponents of the Aristotelian standard approach) and the Unity Challenge (which questions why all the proposed diverse functions of *phronesis* should be carried out by a single character trait rather than a multiplicity of traits) [9].

The Arbitrariness Challenge states that people who support the standard model in general cannot agree on its contents in particular, i.e. its specific functions. But how can one then provide a justifiable list that ascribes certain functions to practical wisdom, and the remaining functions to the individual moral virtues or other character traits [9]? In his earlier work, Miller cites over a dozen examples of philosophers who ascribe different functions to *phronesis* [12]. In our view, most of this apparent dissensus comes down to the vagaries of ordinary language. Relevant terms, such as ‘reflection’, ‘deliberation’, and ‘integration’, are notoriously vague and can be expressed in numerous ways. Although space does not allow us to spell this out here, we believe that most, if not all, of the expressions that Miller cites could fall under one or more of the four main functions of *phronesis* in the standard model that we listed earlier—and none of those functions encroaches upon the specific content/aim of moral virtues such as compassion or honesty. Even proponents of the Aretai model (who do not want to have any truck with the standard Aristotelian model) come up with more or less the same functions [10]. We also notice this broad conceptual acceptance in the context of medicine [3, 27]. Moreover, we can see these core dimensions manifested in the way physicians use moral deliberation to respond well to the challenges of patient care [18, 28, 29].

This relative consensus should not be a source of great surprise. It stands to reason that if one wants to tackle a moral quandary, be it in a medical context or daily life, one needs to: (a) understand what it is about, (b) be calm enough to deliberate about it, (c) have some sense of what one is aiming for, and (d) be apt at going through the relevant checks and balances in order to reach a credible outcome. Krettenauer argues that only (c) and (d) should be considered components of practical wisdom and that (a) and (b) are better seen as preconditions [30]. This is a fair conceptual suggestion but carries little practical import, for if (a) and (b) are also *necessary conditions* for practical wisdom to do its work, then they will also need to be included in any putative operationalization and measure of the construct. These general considerations regarding Miller’s Arbitrariness Challenge notwithstanding, what matters in the end is not how different theorists happen to conceptualize the functions or components of *phronesis*, but rather how those ‘behave’ and interact empirically, as we explain in the fourth section below.

However, even if some basic non-arbitrary list of *phronesis* functions can be established, we next enter that part of the conceptual terrain where the standard model encounters its severest trial. In proposing the Unity Challenge, Miller suggests that “[i]f there are multiple functions ascribed to practical wisdom, then the challenge is to explain how they would all be carried out by a single character trait, especially given how diverse the functions tend to be” [9, p. 6]. On his account, practical wisdom is not an integrated, unified capacity but rather a questionable way of summarizing multiple functions that are divisible into several distinct capacities. This is the basic challenge underpinning his eliminativism, which is defined as follows: “We should reject the appeal to one intellectual virtue, practical wisdom, which is supposed to carry out some or all of the functions associated with practical wisdom. Instead, for each function, we can appeal to a distinct trait that corresponds to that function” [9, p. 6].

We would argue that this assessment mistakenly attempts to reduce an inherently complex, multidimensional capacity which cannot be fragmented without denaturing the interdependence of its several dimensions. Miller recognizes as much, given his awareness that his conceptual fragmentation would deprive practical wisdom of the multidimensionality which the standard model holds to be essential.

We believe Miller’s inclination to isolate these functions through fragmentation deprives them of significance which depends on interrelationships. As supporters of the standard model, we maintain that a meaningful concept of practical wisdom needs to avoid fragmentation because practical wisdom is inherently a capacity that entails a synergic integration: of ends, means, perception, context, virtues, deliberation, reason-guided emotion, and motivation. Through this integration practical wisdom manifests the multidimensional responsiveness that is needed to make excellent moral decisions in concrete circumstances and in real time. It seems to us that Miller’s willingness to fragment the idea of practical wisdom into independent functions does not sufficiently appreciate the need for holistic responsiveness in moral decision-making. We offer two ways of parrying Miller’s eliminativism in the fourth section below (from analogy and from psychometric data). In the remainder of the present section, we offer some more general preliminary reflections.

It is notable that practical wisdom is not alone among virtues in manifesting multiple dimensions. Take, for instance, the virtue of compassion which comprises four interrelated capacities: recognizing suffering, understanding and empathizing with the sufferer, tolerating uncomfortable feelings, and being motivated to act to alleviate suffering [31, 32]. Operationalized in these ways, compassion is a response to suffering which bears some resemblance to practical wisdom by entailing multidimensional capacities of perception, emotion, motivation, and action. This multidimensionality resonates with everyday experience. Regarding compassion, one can recognize when good intentions are not accompanied by accurate perceptions, or when tender feelings are not followed by caring actions. Regarding practical wisdom, one can see its absence when strategic thinking is not accompanied by good goals, or when good goals are not accompanied by accurate perception of circumstances.

Another way to increase appreciation for the multidimensionality of practical wisdom is to highlight how the multiplicity of its integrated functions contrasts with the singularity of decision and action. Here we note a parallel between the multidimensionality of practical wisdom and the multiplicity of values in ethical decision-making, the latter of which has been articulated by Thomas Nagel as a problem of moral fragmentation [33]. Nagel argues that moral value is plural, but moral decisions are singular, which makes it hard to reconcile incommensurable moral claims when choosing one course of action over another. This occurs (on his account) because the moral life is burdened by an insoluble tension derived from six types of basic moral value: specific obligations, rights, utility, perfectionist ends, private commitments, and self-interest.

Setting aside matters of epistemology and metaphysics that might provide a prioritization scheme for the moral life, we would point out a parallel between Nagel’s idea of fragmented values and Miller’s idea of fragmented capacities. In fact, their similarity may reflect more than a parallel, if the multidimensionality of practical wisdom is seen (in part) as a response to the multiplicity of values that ethical deliberation entails. Relating the singular nature of action to the multidimensionality of both practical wisdom and moral value directly relates to medicine as a practical field in which deliberation often focuses on singular courses of action in the face of multiple goods and values. Medical decision making must often respond to situations that involve multiple reasonable options, or no appealing options at all. But a single course of action must nevertheless be chosen—whether that be to intervene (and if so how), wait and watch, withhold an intervention, withdraw an intervention, or shift the goals of care. Practical wisdom manifests the ability to respond ethically and competently to the singular nature of such decisions. It does so by being a holistic, integrated capacity that meets the need for a single, coordinated action in the face of multiple options and values.

To illustrate, consider the challenge of navigating treatment disagreements, such as when a patient or family insists on a potentially inappropriate or futile treatment [34]. Or consider the reverse situation, when a patient refuses a potentially life-saving treatment that is strongly recommended by a physician [35]. Such scenarios highlight the interdependence of the multiple dimensions of decision making—i.e., diagnosis, prognosis, tests, treatments, goals of care, burdens of treatments, probabilities of outcomes, and the moral principles and virtues that guide person-centered care [3, 36]. These dimensions of decision-making must be taken together and cannot be handled effectively if they are fragmented and treated separately.

Medical practice is justifiably renowned for posing moral challenges that require the integrating capacity of practical wisdom. These challenges regularly arise because patients are persons, illness is complex, and medicine is a practice that depends on moral foundations about which people may disagree. Take, for instance, the challenge of providing compassionate care for patients who are suffering toward the end of life. There can be deep disagreements about the meaning of compassion and whether it can be legitimately expressed through physician-assisted suicide and euthanasia [37, 38]. Such debates raise questions about how the virtue of compassion must be directed by moral reasons if it is to avoid malevolence [39]. The deliberative work of practical wisdom provides such direction by integrating moral virtues and principles within a specific context, along with relevant contextual information.

The preceding remarks focus on the unity of practical wisdom as a multidimensional virtue. But it should also be noted that practical wisdom does not “swallow up” the moral virtues, as suggested by Miller’s Subsumption Challenge [9]. Rather, it engages, directs, prioritizes, and integrates the moral virtues which remain distinct from but intimately related to practical wisdom. In suggesting that the standard model of practical wisdom ends up denying the independence of the moral virtues, it is our impression that Miller neglects the possibility that the multidimensionality of practical wisdom can stand comfortably alongside the moral virtues. Relatedly, it seems that Miller also neglects the common observation that a person can give genuine evidence of possessing a moral virtue and still fail to apply it wisely in real-life circumstances. Think, for instance, of a compassionate person who has kind intentions but lacks practical wisdom and therefore unintentionally aggravates suffering in the attempt to alleviate it. Unwise actions motivated by compassion can make a bad situation worse. A more general response to the Subsumption Challenge is that even when there is clear virtue conflict (say, between honesty and compassion) and *phronesis* has been called upon for adjudication, the primary moral motivation still comes from the underlying moral virtues. One wants to be just because of one’s virtue of justice and honest because of one’s virtue of honesty, not because of *phronesis*. What *phronesis* adds in terms of moral motivation is at best a secondary motivation to make decisions that are in line with one’s overall moral identity and thus to try to reconcile virtue conflicts if possible. Can one be honestly compassionate or compassionately honest in the given situation, or does one of the two virtues have to be fully overridden?

We consider psychologist Dan Lapsley’s version of the Unity Challenge [13] more compelling and challenging, as he does not redirect the different functions of *phronesis* back to the moral virtues but actually specifies distinct psychological capacities (with rich research bases to back them up) that can replace each of the proposed *phronesis* functions, for example with “metacognitions” supplanting the integrative/adjudicative function. What Lapsley contests is thus not the alleged arbitrariness of the functions but rather their construal in a philosophical language that speculatively and cavalierly ignores decades of psychological research into the intricacies of human decision-making—and on top of that the Aristotelian obsession with fitting all of those under a single conceptual umbrella. As previously argued [14], the problem with Lapsley’s suggestion, however, is that psychological constructs are to a great degree *functionally* defined. For example, if constructs like grit and conscientiousness behave empirically in the same way, then one or the other is treated as redundant (a jangle fallacy). The next section outlines how practical wisdom can be treated as a molar property of interconnected factors on functional grounds, this time using empirical argumentation.

## Arguments from analogy and psychometric analysis

In an earlier response to Miller’s skepticism about the need for a multidimensional, integrated concept of practical wisdom, one of the present authors utilized the sport of decathlon as an analogy to illuminate the unique contours of *phronesis* as a synergizing meta-virtue [14]. According to some of the sports literature, it is a fallacy to think that the structure of and training for the decathlon can simply be reduced to that of the ten individual sports combined. Rather, decathlon has a ‘logic’ or 'philosophy’ of its own. Not all athletes highly competent in a number of different sports (and even superstars in a single sport) are cut out for becoming decathletes. They need to possess a certain psychological agility and capacity for frequent switching of strategies and approaches to succeed in this the most grueling of sports. The synergic capacities needed for decathlon have to do with all-roundedness, balance, and strategy-switching, where expertise in the individual ‘events’ (as decathletes refer to the ten sports) is only of limited value. This previous article singled out 17 specific sub-areas of analogy between decathlon and *phronesis*, foregrounding the unique unifying and synergic qualities of both.

In that previous paper it was acknowledged that analogies have limitations in making substantive philosophical points, and that analogies can at best offer suggestive value and provoke food for thought by shifting burdens of proof. It is worth making two additional caveats about the applied strategy from analogy. The first is that, despite the references offered by Kristjánsson and Fowers to the sports literature [14], we have realized that there are prominent figures within sport who reject the idea of a unique rationale for decathlon and consider it a more or less arbitrary collation of different discrete ‘events.’ To put this point somewhat irreverently, we could say that there are those within sports who would resonate with Miller’s skepticism and might be considered ‘decathlon eliminativists.’

More significantly from a philosophical perspective, this particular analogy may have conceptual limitations. Someone could plausibly argue that the multiple features of decathlon represent a manufactured multi-sport, rather than an organically integrated activity. Of greater concern than the arbitrary number of ‘events’ making up the ten is a concern about the divisibility of the activity (notwithstanding the unity of the actor, whose goal is to win a competition). Here an objector could reasonably see a difference between the idea of integrated complexity (a single multidimensional activity, as in *phronesis*) versus that of diversity (multiple activities concatenated in sequence, as in decathlon).

This shortcoming invites consideration of a more fitting analogy. Consider, for example, the multiple-dimensions-in-one activity of playing the violin. This entails a single but complex activity requiring multiple capacities encompassing the simultaneous integration of: (1) synchronously but disparately engaged left and right hands (each with their own multiple techniques), (2) auditory perception (intonation, tone, tempo), (3) feeling (musical phrasing), and (4) rational interpretation (when performing a composed work). The ‘complete’ violinist brings all these dimensions together at the same time, whereas the ‘incomplete’ violinist may be technically strong in some areas and weak in others, or technically strong in all areas but lacking in musicality. The former would be analogous to the *phronimos* (a person whose life is characterized by *phronesis*).

The limitation with the earlier analogy is that the decathlon example does not do justice to the integrated nature of real-world challenges that call for practical wisdom. These challenges are typically complex tangles that cannot be teased apart and addressed one at a time sequentially like the separable events of a decathlon. Nevertheless, even the alternative violin analogy still remains just an analogy, with a limited capacity to undermine philosophical arguments. Let us, therefore, turn to a completely different kind of argument, one based on psychometric data.

Psychological constructs are not metaphysical declarations; they constitute empirical hypotheses about how people actually think, feel, and behave. To characterize a theoretical concept such as *phronesis* as a psychological construct is to make a testable claim: that there exists a coherent, measurable pattern in how people respond to items designed to capture that phenomenon. In modern psychometrics, there are well-established standards for evaluating whether a construct is psychologically ‘real’. It is not enough to claim that a construct is conceptually meaningful or theoretically elegant. It must also show up in the empirical structure of actual responses from real people. Constructs, in this sense, are not perspectives; they are patterns.

With this in mind, McLoughlin et al. approached the measurement of *phronesis* not as a matter of interpreting Aristotle, but as a matter of testing whether the phenomenon that Aristotle described and that modern virtue theorists have elaborated actually holds together as a measurable psychological entity [8]. The authors were mindful that Aristotle, for all his conceptual brilliance, operated with relatively blunt theoretical tools. His division of the soul and his functional characterization of virtue provided a rich philosophical framework, but not necessarily a fine-grained psychological one. It was entirely plausible that, upon close empirical inspection, *phronesis* would turn out to be either simpler or more complex than Aristotle supposed.

To begin with, McLoughlin et al. used exploratory factor analysis (EFA), a method that identifies how people’s responses to a wide range of items naturally group together [8]. For example, if there are groups of questions such that responses to one of those questions are indicative of responses to the rest, one can say that the group of questions are ‘measuring the same thing’, functionally speaking. At the same time, other groups of questions may have their own patterns of covariation, and one might then say they are measuring something else. In this way, EFA does not begin with assumptions about how many factors there are or what they should be. It lets the structure emerge from the data. It is a discovery process, not a confirmation of theory.

To illustrate the logic more clearly, consider the following item format from the Short *Phronesis* Measure. Participants might read a short scenario such as: *“Your best friend reveals that they cheated on an important exam and asks you to keep it a secret”.* They are then asked: *“You need to decide what to do in this situation. Which of the following are most relevant to your decision?”* The response options present four virtues—say, honesty, loyalty, generosity, and moderation. Two of these may be correct (in this case, honesty and loyalty), so the item yields an accuracy score ranging from 0 to 2. Now consider what happens across a large set of such items. It is entirely possible that participants who are adept at recognizing when honesty is relevant across scenarios are no more or less likely to recognize when kindness is relevant. If this pattern holds, then the two capacities behave independently. Knowledge of a person’s accuracy on honesty-related items would tell us nothing about their performance on kindness-related items. In such a case, we would treat these as distinct abilities. However, the data may reveal a different pattern. Suppose that participants who perform well on honesty items also perform well on integrity items, and those who perform poorly on one tend to perform poorly on the other. In that case, the two capacities co-vary. Once one knows how well a person recognizes honesty, one can largely predict how well they recognize integrity. Psychometrically speaking, these are not two functionally independent abilities but two expressions of the same underlying capacity. They function as a single dimension.

EFA rests on this logic. It does not begin by assuming how many abilities there are; instead, it examines the patterns of covariation among items. If clusters of items rise and fall together across participants, they likely reflect the same underlying factor. If other items vary independently, they may represent separate factors. What begins as a conceptual distinction between virtues becomes, through these covariance patterns, an empirical question about how many psychologically distinct abilities actually operate in practice. In this sense, factor analysis is a way of letting the data reveal the architecture of the abilities one sets out to measure, making questions of how many abilities, personality factors (etc.) empirically tractable. While there are many plausible philosophical accounts of what different psychological capacities might involve, plausibility alone does not discharge the empirical burden of proof. Without such a burden, theoretical distinctions can proliferate indefinitely, each resting on conceptual elegance rather than observable reality. One thinker may divide the mind into three faculties, another into seven, and a third into forty-nine; yet in the absence of evidence, these taxonomies remain speculative architectures rather than demonstrated truths. It is not the task of science to refute every untested proposal. Rather, science advances by examining what can be measured and by allowing patterns in the data to arbitrate among competing claims. In this respect, EFA provides a disciplined empirical alternative to purely philosophical debate over the components of *phronesis*, grounding the discussion in observable psychological structure rather than conceptual preference alone.

In theory, the EFA used by McLoughlin et al. could have supported a number of possibilities. It might have revealed a single factor, consistent with a virtue monistic view, such as the earlier-mentioned Aretai model. It might also have unproblematically revealed four factors, aligning fully with the standard model. But it revealed neither. Instead, ten distinct but interrelated factors emerged. These reflected a more differentiated and psychologically nuanced structure than previous models had allowed for.

To ensure that this ten-factor solution was not simply a quirk of the initial dataset, McLoughlin et al. then conducted confirmatory factor analyses (CFAs) using two large (*N* = 4000), independent, and representative (census-matching) samples, one from the United Kingdom and one from the United States. In this phase, the goal was no longer to discover structure but to test whether a previously observed structure was confirmed in a new data set. This is a key step in establishing whether a psychological model is stable and generalizable. In both samples, the ten-factor model demonstrated good fit. That is, people in two entirely new populations responded to the items in ways that consistently grouped into the same ten dimensions. This provided robust evidence that *phronesis*, when measured psychometrically, is not a single simple trait or a neat four-component hierarchy, but a ten-component structure. These findings, by themselves, already indicated that the traditional four-component model was too conceptually coarse to capture the de facto complexity of the construct.

Nevertheless, the researchers wanted to give the standard model the benefit of the doubt. Using hierarchical CFA, they tested whether the ten empirically derived factors could be grouped into four higher-order factors, in line with the standard model. In hierarchical models, ‘fitting into’ a higher-order factor means that people’s responses to the ten dimensions are strongly shaped by broader domains. For example, if moral perception were a true higher-order construct, then all its associated sub-components should co-vary closely enough to be treated as manifestations of a single underlying capacity. But the data did not support this. The ten factors did not load cleanly onto the four standard-model domains. Even when forced into that structure, the model showed less-than-ideal fit. If the four domains were boxes into which the ten components might empirically fit, the overarching factor was the cupboard meant to contain the boxes. But the cupboard was ill-fitting; the boxes did not stack neatly.

This may have seemed to be really bad news for the standard model. However, on closer theoretical inspection, the ten elicited factors fall neatly as sub-functions under the original four functions; so the *logical structure* of the standard model holds up well, if it is understood simply as a taxonomical model for the aims that *phronesis* is meant to achieve in our moral lives (Table 1).

**Table 1:** Components of the older Aristotelian phronesis model (APM) and neo-APM

APM component	Neo-APM component	Definition
Moral perception	Virtue identification	The objective ability to identify virtues relevant to a scenario in which what a person does affects their character
	Situational moral relevance	The objective ability to correctly determine when what someone does in a given situation affects their character
	Situational moral irrelevance	The objective ability to correctly determine when what someone does in a given situation does not affect their character
Moral emotion	Emotional regulation	The subjectively assessed ability to handle one’s emotions in an emotionally charged scenario
	Positive moral emotion	The self-assessed propensity to feel good about oneself when one does the right thing
	Negative moral emotion	The self-assessed propensity to feel bad about oneself when one does the wrong thing
Moral blueprint	Moral self relevance	How strongly individuals experience moral considerations as personally significant
	Aspired moral identity	The degree to which a person has articulated and is working towards their “best moral self”
Moral adjudication	Moral deliberation	The tendency to seek out relevant information and reflect carefully before making a decision in a morally significant situation
	Moral integration	The propensity to bring all morally relevant considerations into view when making a decision—including emotions, identity, context, and competing concerns—rather than ignoring or sidelining something important

What the analysis shows, however, is that the mind does not appear to operate neatly along those logical categories; it appears to divide the tasks of the four aims up into bundles that are collaborated upon in different areas of the mind, much as, for example, a marketing department in a company might take over some tasks that are logically more in line with the jobs specs of the sales team.

These findings also have implications for the deeper question of *phronesis* as a psychological capacity. One could imagine rescuing a unified interpretation by positing a single overarching ability that coordinates the ten components. Conversely, one might try to argue that the components are largely independent skills with no meaningful unity. A bifactor model, which specifies one general factor alongside several specific components, would have been an appropriate structural representation of a unified *phronesis* capacity if such a capacity existed at the level of shared variance. However, the bifactor model did not fit the data. This rules out a simple monistic interpretation, but it does not vindicate Miller’s view either. The components are clearly not independent, because their covariation forms a systematic pattern. Rather than thinking hierarchically, with *phronesis* governing the virtues from above, it may be more accurate to see *phronesis* as emerging from a network of interrelationships between psychological capacities. In this framework, *phronesis* is not something that stands entirely above the virtues and directs them, like a conductor over an orchestra. Instead, it is the dynamic configuration that reflects a person’s capacity for virtue-relevant traits that interact, reinforce, and calibrate one another in context.

Network analysis allows us to model such relationships directly. It allowed McLoughlin et al. to treat each of the ten factors as a ‘node’ and map the connections between them. In network analysis, some nodes are more central than others, meaning they are more strongly and widely connected within the network. Centrality, in this context, refers not to conceptual importance but to structural influence. A central node is one where its variation (high or low) is associated with variation in many other nodes in the system. It is, in effect, a hub through which other capacities appear coordinated. The network analysis employed by McLoughlin et al. revealed something surprising. The most central traits were not the moral adjudication capacities, i.e., they were not capacities that play the crucial ‘coordinating’ role. Instead, the two most central nodes were the ones falling logically under moral identity or ‘blueprint’: Moral Self-Relevance and Aspired Moral Identity. The former refers to the degree to which a person sees morality as central to their self-concept, i.e., whether moral values feel personally meaningful and psychologically salient. The latter refers to the extent to which a person has a clear vision of the kind of moral person they want to become and is actively striving toward that ideal. These are both identity-based dispositions. They are about who one takes oneself to be, and who one is trying to become. These findings carry significant lessons for ethics education, for instance within medicine, as it seems natural to begin the process of education with the most central nodes: the moral identity of the student as a person and as an aspiring physician.

Importantly, however, network analysis places emphasis on the co-dependency of factors rather than treating them as isolated traits. Consider, for example, a person with an extremely high level of Aspired Moral Identity, such that their sense of who they ought to be directs both their day-to-day conduct and their long-term life aims. If that same person scores poorly on Moral Deliberation, they may lack the inclination to question whether they possess all the relevant facts before acting in the name of that identity. It is not difficult to see how such a configuration could give rise to dogmatism or zealotry, dispositions that run counter to flourishing. More importantly, the phenomenological meaning of Aspired Moral Identity itself would change under these conditions. When an identity is not tempered by deliberation, it ceases to function as the same psychological construct. It becomes something more rigid, less reflective, and potentially more dangerous. The meaning of each node in the network therefore depends, in part, on the configuration of the others. An everyday analogy may clarify the point. We have a concept of ‘the ground’, but when that same surface appears within a particular arrangement of walls, doors, and windows, it becomes ‘the floor’. The physical substrate has not changed, but its functional meaning has. Likewise, a psychological capacity can take on a different character depending on the structure in which it is embedded. Network analysis therefore highlights varying degrees of co-dependency among component factors, with *phronesis* emerging as the homeostatic coordination of the network’s nodes rather than as a single, self-contained trait, nor as merely the sum of its parts. In this view, practical wisdom is less like a solitary pillar and more like a system of interlocking beams: its strength depends not only on the parts themselves, but on how they hold one another in place.

Centrality also matters, conceptually, in other ways besides. For example, it offers a stopping point for what might otherwise be an infinite regress. If flourishing is constituted (partly) by virtuous behavior, and virtuous behavior is caused by the virtues (i.e., contextually invariant properties of the individual), and the virtues are coordinated by *phronesis*, then one might reasonably ask: what co-ordinates *phronesis*? What determines when *phronesis* succeeds or fails? The network model answers this by showing that what most powerfully shapes the system—what sits at its center—is moral identity. *Phronesis* emerges from how virtue-relevant traits interact in a person whose moral identity is psychologically active and motivationally engaged.

Thus, this empirically grounded version of the Aristotelian standard model reconceptualizes *phronesis* not as a single faculty or a skill (*techné*) or expertise, nor as a mere collation of independent capacities, but as a unified, synergic network structure whose central anchors are identity-related. The network nodes are dispositional orientations: relatively stable, identity-forming elements of character. In this light, *phronesis* is best understood as the dynamic configuration of moral capacities within a person whose identity is morally anchored. It is not a conductor standing above an orchestra; it is the interplay between instruments, with certain themes running through the whole. This is not a loss for virtue ethics or the standard model of *phronesis*—it is a clarification. The analysis of the empirical data did not make *phronesis* redundant. It just helped us to understand it in more detail than would be possible with philosophical argumentation alone.

## Grounds for consensus about core dimensions of practical wisdom

We have argued that it is uncontroversial to maintain that Aristotelian accounts of practical wisdom broadly agree that it is a virtue which facilitates excellence in ethical decision-making by integrating core dimensions. These dimensions concern the ability to recognize and employ the best means to achieve good and worthwhile ends [25, 26]. We have shown that there is considerable consensus concerning the logical contents of these core dimensions, which include moral perception of context, moral perception of goals (proximate and ultimate), moral deliberation and integration, motivation, and harmonization of moral reason and moral emotion within a virtue ethics framework.

It is clear from Aristotle that practical wisdom has to do with moral deliberation about means and ends in order to achieve what is good through truthful rational action in particular cases [15]. This ability of moral deliberation may be understood as a disposition to make right moral judgments, which is a practical intelligence that underlies all the moral virtues [40]. While Aristotle would say that practical wisdom focuses more directly on means, while virtue writ large focuses more directly on ends, the relationship between these two aspects leads naturally to summary statements such as that practical wisdom is the “ability to judge the good and bad ends for man and to choose the actions appropriate for securing those ends in the particular circumstances of life” [41, p. 173], or that practical wisdom allows one to grasp “what one ought to do, all-things-considered, in particular situations” [42]. The *telos*-orientation of practical wisdom invites one to see it as an “aiming” virtue because it identifies, through perception and deliberation, one or more good ends to be achieved by means of the actions it guides, all of which manifests the “intrinsically motivational character” of practical wisdom as it is drawn to and driven by a desire to accomplish what is good [43].

While we have acknowledged that there can be diverse ways to name and apportion core dimensions of practical wisdom and that these different ways do not carve nature (here, of the human mind) at its joints, we believe that most theorists of practical wisdom have nevertheless been dealing substantively with the same core content (cutting the same cake, as it were). Hence our assessment that there is a fundamental consensus. If one keeps to a reasonably general level of description, one can find ample evidence of agreement for these core dimensions [3, 44, 45].

Given the enduring historical influence of Aristotelian (and later Thomistic) traditions on contemporary understandings of practical wisdom, one should not be surprised by the presence of such consensus. Moreover, one should not be surprised to see evidence of these core dimensions in discussions of practical wisdom, or prudence, in the specific contexts of medicine and bioethics. In medicine, practical wisdom is described as the capacity to guide one toward “a right way of acting in difficult and uncertain circumstances for a specific end, i.e., the good of a particular patient who is ill” [46, p. 250]. In fact, practical wisdom has been described as “medicine’s indispensable virtue” because it disposes one to “attain the truth for the sake of action” by identifying the means that “are most appropriate to the good in particular circumstances” [47, p. 84].

Practical wisdom provides a capacity for deliberation that is of central importance in the health professions because substantive clinical decisions require weighing of alternatives amid stress and uncertainty, with the ability to know how to untangle apparent conflicts among the virtues, understand their interrelationships to one another, and select the most appropriate means by which to fulfill the goals of the healing relationship [48], thereby achieving a “right and good decision” for a particular patient [23]. Practical wisdom provides the capacity for good judgment that coordinates and integrates relevant variables in patient care so that a clinician discerns and prioritizes what is important, both factually and ethically, to determine the best course of action and way of interaction, all things considered [49]. In bioethics, some may refer to practical wisdom as the virtue of discernment, by which clinicians know how to apply ethical principles in a particular circumstance and bring “sensitive insight, astute judgment, and understanding to bear on action” so that they can respond with the right intensity of feeling, in the right way, at the right time, with the right balance of reason and desire [50].

In addition to conceptual evidence of consensus about core dimensions of practical wisdom in medicine, it is becoming increasingly clear from recent empirical studies that healthcare students and healthcare professionals endorse these core dimensions of practical wisdom. A study of 80 medical students and 22 nominated physicians demonstrated that participants understood practical wisdom as being broadly applicable to medicine and having multiple dimensions that included being deliberative, goal-directed, context-sensitive, and involving motivation, ethics, integrity, and interrelationships with many moral virtues [51]. A study of 19 internal medicine physicians showed that wisdom in medicine was understood to entail the deliberative integration of medical knowledge, ethics, perception of context and circumstances, and goals [52]. And a study of 31 surgeons found that they understood surgical wisdom as practical wisdom that facilitates context-dependent and value-interacting choices; these authors also noted how their findings support the standard model via the ability to perceive competing values, select values to act upon, act in a manner congruent with one’s identity, and respond with appropriate emotions [53].

In light of such evidence of conceptual and empirical consensus, it seems fair to say that among proponents of the standard model of practical wisdom, and among those who write and gather data about practical wisdom in medical settings, there appears to be a substantial degree of agreement about the core dimensions of practical wisdom. This consensus encompasses capacities that include patient-centered deliberation directed to goals of health and flourishing, perception of particular contexts and circumstances, integration of moral virtues and principles, harmonization of reason and emotion, and a motivation to promote the patient’s good.

To sum up, in this article we have offered a series of arguments that paint a plausible picture of practical wisdom as (1) a meta-virtue with multiple dimensions that is nevertheless not reducible to those several dimensions, (2) a virtue that augments and integrates the moral virtues, and (3) a virtue whose core dimensions enjoy broad consensus and can be empirically verified. The idea that *phronesis* is a unified and unifying intellectual meta-virtue that guides the moral virtues is not new. It has survived for more than 2300 years, and we believe the reasons put forward here indicate that *phronesis* will continue to endure as a meaningful, multidimensional concept because it reflects the moral deliberation we need as we respond to practical challenges in life and medicine.
